# The Golgi apparatus: Site for convergence of COVID-19 brain fog and Alzheimer’s disease?

**DOI:** 10.1186/s13024-022-00568-2

**Published:** 2022-10-21

**Authors:** Yanzhuang Wang, Sam Gandy

**Affiliations:** 1 Department of Molecular, Cellular, and Developmental Biology and Department of Neurology, University of Michigan, 48109 Ann Arbor, MI USA; 2 Departments of Neurology and Psychiatry and the Mount Sinai Alzheimer’s Disease Research Center, Icahn School of Medicine at Mount Sinai, 10029 New York, NY USA; 3 The James J Peters VA Medical Center, 10468 Bronx, NY USA

**Keywords:** Alzheimer’s disease, Autophagy, Brain fog, Golgi, Glycolipids, Glycosylation, GRASP55, GRASP65, Heparan sulfate proteoglycans, Intracellular trafficking, Myelin, O-GlcNAcylation, Phosphorylation, SARS-CoV-2 infection, Secretion

## Perspective

The ongoing COVID-19 pandemic has affected millions of people worldwide. One interesting observation, revealed by a few recent studies [1–3], is that SARS-CoV-2 infection can apparently cause an Alzheimer disease (AD)-like neuropathological phenotype as well as clinical “brain fog”. This raises an interesting question: “where and how might the molecular pathways underlying SARS-CoV-2 infection and AD converge?”

**Fig. 1:**
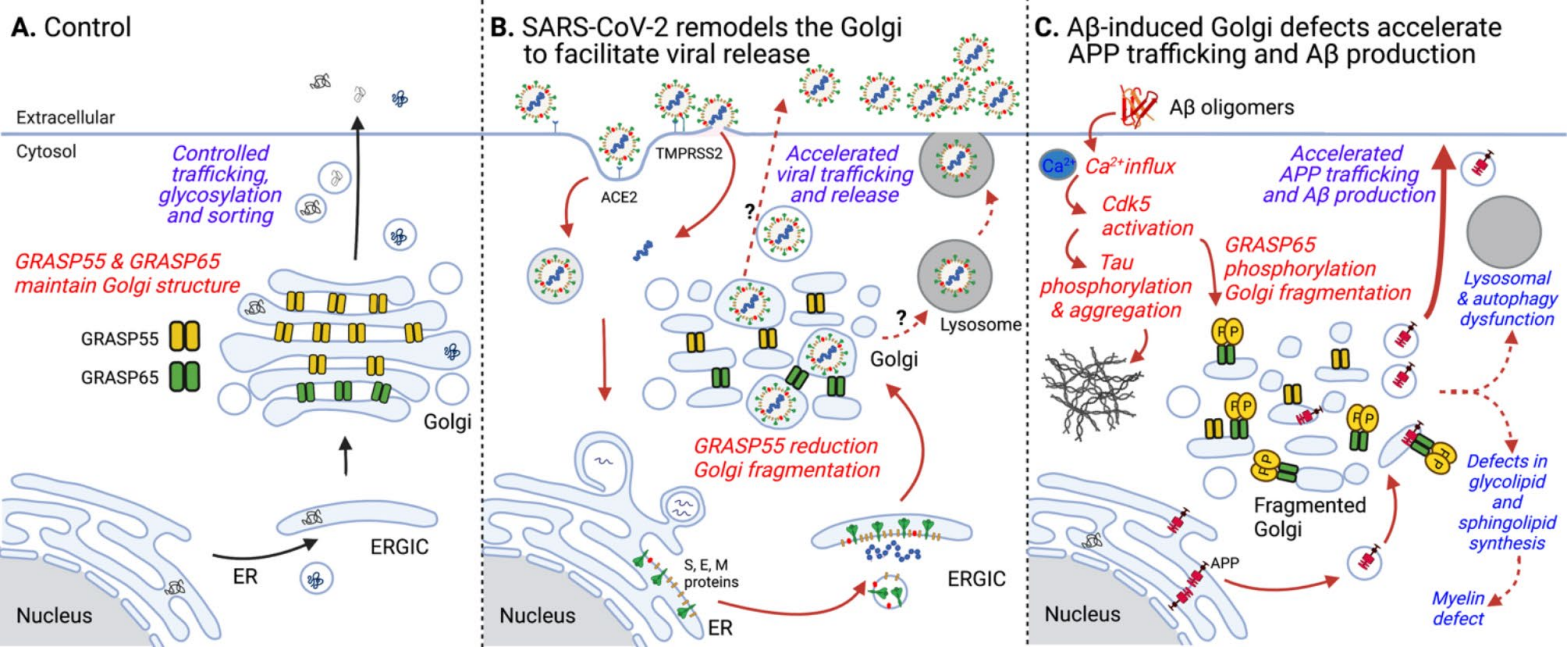
**Golgi defects accelerate SARS-CoV-2 virus maturation in COVID-19 and Aβ production in AD. (A)** In healthy cells, Golgi structure formation serves as a quality control mechanism for protein glycosylation and sorting. When Golgi cisternae are fully stacked, which is mediated by Golgi structural proteins including GRASP55 and GRASP65, vesicles can only form and fuse at the rims. This slows down trafficking, but enforces accurate glycosylation and sorting [4]. **(B)** In COVID-19, SARS-CoV-2 infection causes Golgi fragmentation possibly by downregulating GRASP55 expression, which may accelerate virus maturation and release. **(C)** In AD, Aβ oligomer accumulation leads to the influx of Ca^2+^ ions; increased cytosolic Ca^2+^ activates calpain to cleave p35 to p25. p25 then activates cdk5 for GRASP65 phosphorylation. GRASP65 phosphorylation results in Golgi fragmentation, which, in turn, accelerates APP trafficking and increases Aβ production [9]. In addition, Golgi defects also cause autophagy and lysosomal dysfunction [4] and impact the synthesis of glycolipids [4, 7], major components of the myelin sheath.

SARS-CoV-2 is an enveloped virus whose replication, assembly and release rely on the secretory pathway of the host cell. Three non-structural proteins (Nsp3, 4, 6) remodel the endoplasmic reticulum (ER) to form double-membrane vesicles (DMVs) that facilitate viral RNA replication. Virions are assembled and bud into the lumen of the ER-Golgi intermediate compartment (ERGIC) and Golgi where the spike protein is glycosylated and cleaved by furin. The Golgi is a key cellular structure for membrane trafficking, processing, and sorting. To perform these functions, it needs to form a multilayer stacked structure by two Golgi stacking proteins, GRASP55 and GRASP65 (Fig. 1A). Depletion of GRASPs leads to Golgi fragmentation (GF), which accelerates trafficking but causes missorting of lysosomal enzymes, alters protein glycosylation and secretion, and affects other cellular activities such as cell attachment, migration, and growth [4]. SARS-CoV-2 infection triggers GF, which accelerates viral trafficking and release [5] (Fig. 1B). In addition, the spike-ACE2 interaction is enhanced by heparan sulfate [6] whose synthesis is increased by GRASP depletion [7]. In a recent study [2], treatment with serum from SARS-CoV-2-infected hamsters causes a SARS-CoV-2 like phenotype in naive hamsters, indicating that SARS-CoV-2 infection triggers the secretion of unknown molecules into the serum.

GF occurs widely in brain samples of AD patients and can be induced by excessive activation of neurons [8]. Many AD pathologies, including abnormal protein sorting and glycosylation, impaired lysosomal/autophagosomal degradation, increased production of the toxic Aβ peptide, as well as tau phosphorylation and aggregation, can be related to Golgi defects. We have discovered that Aβ oligomer accumulation triggers GF by activating cyclin-dependent protein kinase 5 (cdk5), which, in turn, phosphorylates GRASP65 and causes GF [9]. Subsequently, GF accelerates amyloid precursor protein (APP) trafficking and increases Aβ production (Fig. 1C). Conversely, rescue of the Golgi structure by expressing a phosphorylation-deficient mutant of GRASP65 reduces Aβ production by increasing non-amyloidogenic α-cleavage of APP [9]. Given that cdk5 is a major kinase for tau phosphorylation, this observation links enhanced APP amyloidogenic cleavage with tau phosphorylation.

GRASP55 also regulates the secretion and aggregation of cytoplasmic neurotoxic proteins through autophagy. Under growth conditions, GRASP55 is modified by a post-translational modification that attaches *O*-linked N-acetylglucosamine (*O*-GlcNAc) moieties to serine and threonine residues of nucleocytoplasmic proteins (*O*-GlcNAcylation) and functions as a Golgi stacking protein. Glucose deprivation reduces GRASP55 *O*-GlcNAcylation, allowing it to relocate from the Golgi to the interface between autophagosomes and lysosomes, where it functions as a membrane tether to facilitate autophagosome-lysosome fusion [4]. This increases unconventional secretion of cytosolic neurotoxic proteins such as tau and mutant huntingtin and reduces their aggregation [10]. Therefore, molecular and cellular dysfunctions related to GF may extend beyond the Golgi *per se*, resulting in dysfunction of distal compartments of the secretory pathway including autophagosomes and lysosomes, and impact a variety of cellular activities.

The similarity between COVID-19 and AD in causing GF indicates that the Golgi may be a candidate subcellular locus where the two diseases converge. Both the SARS-CoV-2 spike protein and APP are type I membrane proteins that travel through the secretory pathway. In the Golgi, they are both modified by N- and O-glycosylation and processed by proteases, both of which are impacted by GF. It is possible that SARS-CoV-2 infection may cause GF and subsequently accelerate APP trafficking and processing, thus leading to AD neuropathology [1].

“Brain fog” is a general term used to describe cognitive impairment observed under different circumstances including SARS-CoV-2 infection. SARS-CoV-2 infects all neuronal types [1], and SARS-CoV-2 infection-caused GF may also enhance the secretion of cytokines such as TNFα and IL-1β. While the mechanism of brain fog is currently unknown, one possible explanation is that SARS-CoV-2 infection causes a defect in myelination [3]. The Golgi is the cellular “factory” for sphingolipid and glycolipid synthesis. GF leads to a substantial elevation in the levels of monosialotetrahexosylganglioside (GM1) with concomitant reduction of globotriaosylceramide (Gb3) [4]. While both GM1 and Gb3 play important roles in myelination and function, GM1 has also been shown to enhance Aβ aggregation and toxicity.

In summary, the mechanisms by which SARS-CoV-2 infection impacts brain cells at the molecular and cellular levels are important issues to elucidate, and the appearance of GF may serve as a key starting point. Perhaps it is time to look more deeply into Golgi abnormalities, which may shed light on the connection between AD pathology and clinical dementia. Elucidation of how Aβ fibrils and oligomers cause defects in trafficking and sorting of many proteins in a variety of brain cell types will be required to make sense of the abnormalities in glycosylation, lysosomal function, and autophagy that define the molecular cell pathology of AD. Speculation about the potential role for GF in neurodegeneration and SARS-CoV-2 infection may be more than just an academic exercise. It is conceivable that manipulation of GRASPs and other Golgi proteins may provide novel potential therapeutic opportunities for a host of currently untreatable major human diseases. This *Perspective* raises the possibility that GRASPs *per se*, or GRASP-mimetic molecules or drugs, might be useful in the treatment or prevention of neurodegenerative or other diseases. Efforts at testing this possibility in various cellular and animal models of human diseases are currently underway.

## Electronic supplementary material

Below is the link to the electronic supplementary material.

Supplementary Material 1
