# Supplementary material for: The Golgi apparatus: Site for convergence of COVID-19 brain fog and Alzheimer’s disease?
Source: Mol Neurodegener. 2022 Oct 21;17:67. doi: 10.1186/s13024-022-00568-2 (PMC9587685; doi:10.1186/s13024-022-00568-2)
Supplement: Supplementary file 1 — Supplementary Material 1 [file 13024_2022_568_MOESM1_ESM.pdf]

Icahn School of Medicine at Mount Sinai

Sam Gandy, M.D., Ph.D.
Professor of Psychiatry and Neurology
Mount Sinai Health System Department of Neurology
One Gustave L. Levy Place, Box 1137
New York, NY 10029-6574
Tel: (212) 241-7076
Direct eFax: (267) 389-2800
Email: samuel.gandy@mssm.edu

MOND-D-22-00125R3 September 2, 2022

"The Golgi apparatus: Site for convergence of COVID-19 brain fog and Alzheimer's disease?"
Yanzhuang Wang, Sam Gandy

Lucy Job, Ph.D.
Associate Editor, *Molecular Neurodegeneration*

Dear Dr. Job,

Thanks to you and Hongmei for accepting our *Perspective: "The Golgi apparatus: Site for convergence of COVID-19 brain fog and Alzheimer's disease?*" for publication in *Molecular Neurodegeneration*.

As advised, we are submitting the final accepted version as a new submission. Please see the following pages as a reminder of our conversation by email.

We look forward to seeing our *Perspective* in print in *Molecular Neurodegeneration*.

Sincerely,

Sam Gandy, M.D., Ph.D.
Cell (267) 240-6551

Resubmission of MOND-D-22-00125 & another case...

Hongmei Li <Hongmei.Li@MolecularNeurodegeneration.org>
Thu 9/1/2022 1:10 PM
To: Gandy, Samuel <samuel.gandy@mssm.edu>;Lucy Job <Lucy.Job@MolecularNeurodegeneration.org>

Dear Sam,

For MOND-D-22-00125 resubmission, please do it as if you are submitting a new manuscript but mention in the cover letter that MN's editorial team has approved of this resubmission.

Also, thank you so much for you immediate action in inviting peer-review for MOND-D-22-00373 ("Combined Metabolic Activators Improves Cognitive Functions in Alzheimer's Disease Patients"). It's amazing that you've obtained 7 reviewers for it so promptly! Thank you so much for your effort in sending so many invitations and efficiently recruiting qualified experts for this submission.

Sorry that I forgot to communicate to our editors earlier that our Chiefs implemented a "new" rule about a year or two ago to have n**o more than 5 reviewers per submission**. They requested a minimum of three reviewers per sub long time ago, but added this "no more than 5" because I got 6 reviewers for one particular submission, and our Chiefs decided it was a bit unreasonably too painful for the author team.... From then on, when we obtained more than five reviewers, we would uninvite the extra reviewers to save reviewers' time and make the peer-review more manageable. Now that we have 7 reviewers for this one, could you please pick two to be uninvited? It can be easily done in the system, and Lucy can help with carrying out the procedure if you prefer. Here is the list. If it all the same for you, I might suggest to uninvite JinChao Hou (Marco Colonna's postdoc) for one, as she is supposed to work on an invited review for us, which has been dragged over a long time. She just promised me to submit it by the end of September, and I'd hope she will focus on that task....

Gregory S Day (Reviewer)	Agreed to Review<br>*31 Aug 2022*	Un-assign
JinChao Hou (Reviewer)	Agreed to Review<br>*31 Aug 2022*	Un-assign
Justin Long (Reviewer)	Agreed to Review<br>*01 Sep 2022*	Un-assign
Michael (Mike) Heckman (Reviewer)	Agreed to Review<br>*01 Sep 2022*	Un-assign
Richard Mead (Reviewer)	Agreed to Review<br>*01 Sep 2022*	Un-assign
Samir Abu Rumeileh (Reviewer)	Agreed to Review<br>*31 Aug 2022*	Un-assign
Zhengyang Zhou (Reviewer)	Agreed to Review	

Please feel free to reach out to us if there is anything we can help with.

Thanks again, and
Cheers,

Hongmei

Hongmei Li, Ph.D.
Senior Editor
Molecular Neurodegeneration
http://molecularneurodegeneration.biomedcentral.com/

[ISMND 2022 Conference](#)
Glial and Vascular Contributions to Neurodegenerative Diseases
Athens, Greece
Oct 9 - 12, 2022

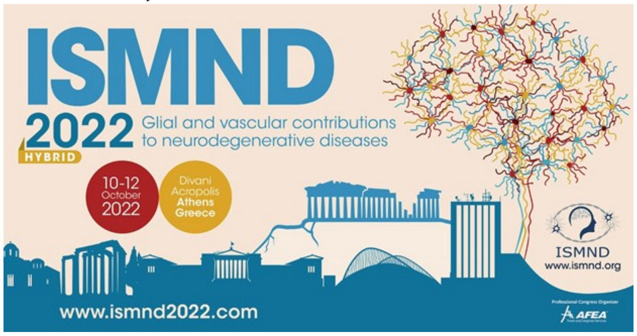

From: Gandy, Samuel <samuel.gandy@mssm.edu>
Sent: Thursday, September 1, 2022 12:19 PM
To: Lucy Job <Lucy.Job@MolecularNeurodegeneration.org>
Cc: Hongmei Li <Hongmei.Li@MolecularNeurodegeneration.org>
Subject: Re: Attention Dr Lucy Job re MOND-D-22-00125

Lucy & Hongmei,

That is fine, but I need some way to upload the accepted final short version.

The final decision of Reject does not permit me to upload the final short version.

Thanks.
Sam

From: Lucy Job <Lucy.Job@MolecularNeurodegeneration.org>
Sent: Thursday, September 1, 2022 11:52 AM
To: Gandy, Samuel <samuel.gandy@mssm.edu>
Cc: Hongmei Li <Hongmei.Li@MolecularNeurodegeneration.org>
Subject: Re: Attention Dr Lucy Job re MOND-D-22-00125

Hi Sam,

We will accept the short-form version without peer-review. However, at this moment, the MN editorial blog has been retired due to the publisher's reformation. We are working with the publishing team to get this back up and running, however, it may take a while. I will let you know if there are updates.

Best wishes,
Lucy

From: Gandy, Samuel <samuel.gandy@mssm.edu>
Date: Thursday, 1 September 2022 at 08:53
To: Lucy Job <Lucy.Job@MolecularNeurodegeneration.org>
Subject: Re: Attention Dr Lucy Job re MOND-D-22-00125

Lucy,

Any update on this before the long (US Labor Day) holiday weekend?

Thanks,
Sam

From: Gandy, Samuel <samuel.gandy@mssm.edu>
Sent: Monday, August 29, 2022 12:44 PM
To: Lucy Job <Lucy.Job@MolecularNeurodegeneration.org>
Cc: yzwang@umich.edu <yzwang@umich.edu>
Subject: Re: Attention Dr Lucy Job re MOND-D-22-00125

Thanks, Lucy.
We will sit tight until you sort this out.
No problem.

Sam

Get Outlook for iOS

From: Lucy Job <Lucy.Job@MolecularNeurodegeneration.org>
Sent: Monday, August 29, 2022 12:41:37 PM
To: Gandy, Samuel <samuel.gandy@mssm.edu>
Cc: yzwang@umich.edu <yzwang@umich.edu>
Subject: Re: Attention Dr Lucy Job re MOND-D-22-00125

Dear Sam,

I've just been informed that in order for you to submit your short perspective with the link to the longer version for the blog, we need to first sort out the editorial blog issues we are currently having. We have a meeting with the publishing team at 2pm EST, so once the outcome of that meeting has yielded more information, I can then let you know how to submit correctly.

I apologise for the inefficiency, but I hope to get this sorted as soon as possible.

Best wishes,
Lucy

From: Gandy, Samuel <samuel.gandy@mssm.edu>
Date: Monday, 29 August 2022 at 11:21
To: Lucy Job <Lucy.Job@MolecularNeurodegeneration.org>
Cc: yzwang@umich.edu <yzwang@umich.edu>
Subject: Re: Attention Dr Lucy Job re MOND-D-22-00125

Thanks, Lucy.
Could you tweak the website so that I can upload the short version?
The final rejection category does not permit submission of a revision.

Sam

From: Lucy Job <Lucy.Job@MolecularNeurodegeneration.org>
Sent: Monday, August 29, 2022 12:19 PM
To: Gandy, Samuel <samuel.gandy@mssm.edu>
Subject: Re: Attention Dr Lucy Job re MOND-D-22-00125

USE CAUTION: External Message.

Dear Sam,

I apologise for the delay. I am pleased to inform you that your appeal has been successful, and we welcome you to submit your perspective (the short version) to MN. We are working with the publishing team to sort out the editorial blog situation, I will keep you updated.

Best wishes,
Lucy

From: Lucy Job <Lucy.Job@MolecularNeurodegeneration.org>
Date: Wednesday, 17 August 2022 at 13:00
To: Gandy, Samuel <samuel.gandy@mssm.edu>
Subject: Re: Attention Dr Lucy Job re MOND-D-22-00125

Dear Sam,

Thank you for sending over these documents. I will send them over to the editorial team. Please give up to one week for the editorial team to reach an outcome.

Best wishes,
Lucy

From: Gandy, Samuel <samuel.gandy@mssm.edu>
Date: Sunday, 14 August 2022 at 14:26
To: Lucy Job <Lucy.Job@MolecularNeurodegeneration.org>
Subject: Attention Dr Lucy Job re MOND-D-22-00125

From: Gandy, Samuel <samuel.gandy@mssm.edu>
Sent: Sunday, August 14, 2022 1:53 PM
To: em@editorialmanager.com <em@editorialmanager.com>
Subject: Attention Dr Lucy Job re MOND-D-22-00125

August 14, 2022

Lucy Job, Ph.D.
Molecular Neurodegeneration

Dear Dr Job,

Please see my attached cover letter and other accompanying documents.
The manuscripts are attached in both Word and PDF formats.

Thanks.
Sam

Sam Gandy, M.D., Ph.D.
Professor of Neurology and of Psychiatry
Mount Sinai Endowed Chair in Alzheimer's Disease Research
Chief, Division of Cognitive and Behavioral Neurology
Director, Mount Sinai Center for Cognitive Health
Icahn School of Medicine at Mount Sinai
New York NY 10029
and
James J Peters VA Medical Center
Bronx NY 10468

212-241-7076 Neurology FPA7 (5 East 98th St) Voice Line
